# Resolving relaxometry and diffusion properties within the same voxel in the presence of crossing fibres by combining inversion recovery and diffusion‐weighted acquisitions

**DOI:** 10.1002/mrm.25644

**Published:** 2015-03-02

**Authors:** Silvia De Santis, Daniel Barazany, Derek K. Jones, Yaniv Assaf

**Affiliations:** ^1^ CUBRICSchool of Psychology, Cardiff UniversityCardiffCF10 3ATUK; ^2^ Department of Neurobiology, Faculty of Life SciencesTel Aviv UniversityTel Aviv69978Israel; ^3^ Neuroscience & Mental Health Research Institute, Cardiff UniversityCardiffCF10 3ATUK.

**Keywords:** diffusion tensor MRI, myelin, CHARMED, white matter microstructure, *g*‐ratio

## Abstract

**Purpose:**

A comprehensive image‐based characterization of white matter should include the ability to quantify myelin and axonal attributes irrespective of the complexity of fibre organization within the voxel. While progress has been made with diffusion MRI‐based approaches to measure axonal morphology, to date available myelin metrics simply assign a single scalar value to the voxel, reflecting some form of average of its constituent fibres. Here, a new experimental framework that combines diffusion MRI and relaxometry is introduced. It provides, for the first time, the ability to assign to each unique fibre system within a voxel, a unique value of the longitudinal relaxation time, *T*_1_, which is largely influenced by the myelin content.

**Methods:**

We demonstrate the method through simulations, in a crossing fibres phantom, in fixed brains and *in vivo*.

**Results:**

The method is capable of recovering unique values of *T*_1_ for each fibre population.

**Conclusion:**

The ability to extract fibre‐specific relaxometry properties will provide enhanced specificity and, therefore, sensitivity to differences in white matter architecture, which will be invaluable in many neuroimaging studies. Further the enhanced specificity should ultimately lead to earlier diagnosis and access to treatment in a range of white matter diseases where axons are affected. Magn Reson Med 75:372–380, 2016. © 2015 The Authors. Magnetic Resonance in Medicine Published by Wiley Periodicals, Inc. on behalf of International Society of Medicine in Resonance.

## INTRODUCTION

White matter (WM) is a complex biological medium responsible for carrying information within neural networks. WM pathways comprise a set of axons coated with myelin sheaths to providing optimal impulse propagation along the fibres. To characterize WM, it is essential to be able to characterize independently the two distinct attributes: the axonal features and the myelination.

MRI techniques are invaluable for characterizing WM. Diffusion tensor MRI 1 allows estimation of biomarkers that reflect largely axonal properties, but are also modulated by the myelin content 2, failing to resolve the intrinsic duality of WM 3, 4. To get more biological specificity, different approaches have been introduced to look at myelin and axonal properties individually. The CHARMED approach 5, 6 models water motion inside the axon separately from that outside the axon, providing a proxy measure of axonal density. More recently, this approach has been extended to provide estimates of axonal diameter 7, 8, 9. MRI‐based methods specific for quantifying myelination have also been developed, including multicomponent relaxometry 10, 11 and quantitative magnetization transfer imaging 12, 13.

The challenge, however, for extending these measurements to the whole brain, is that more than 90% of WM voxels contain more than one fibre population 14. Assigning myelin and axonal measures to distinct fibre populations within a voxel, therefore, requires distentangling the aforementioned quantitative metrics from the fibre architectural paradigm. While several methods have been proposed to assign distinct diffusion properties to distinct fibre populations, for example, fibre‐specific axonal density 5, 6, anisotropy 15, 16, and axonal diameter 17, methods developed for quantifying myelin to date provide only a single (i.e., average) myelin content of the voxel, irrespective of the architectural paradigm.

This work is a step forward to address this limitation with a new acquisition and analysis strategy that combines inversion recovery 18 with diffusion tensor MRI 19. For each fibre population within a voxel, a specific longitudinal relaxation time *T*_1_ is extracted by exploiting the orientational dependence of the diffusion‐weighted signal that has been previously inversion prepared.

Simulations with realistic noise models and experimental constraints are run to prove the feasibility of the method on different test systems. Three different protocols are identified and applied:

1 We test our analysis on a sample comprising two excised porcine nerve fibres, characterized by very different *T*_1_ properties 20, crossing at 90 degrees; 2 we develop the protocol to analyse perfused rat brain; and 3 we apply the protocol *in vivo* in the rat model.

## METHODS

### Diffusion‐Weighted Echo Planar Imaging Pulse Sequence

The imaging experiments used an inversion recovery spin‐echo diffusion‐weighted echo planar imaging (IR‐DTI) sequence. The sequence was built by applying an adiabatic 180 inversion pulse prior to the standard spin‐echo diffusion‐weighted sequence. The time between the inversion pulse and the start of the diffusion‐weighted sequence, or inversion time (TI), was changed to span the range of expected *T*_1_ decay. All imaging data were acquired on a 7T Bruker MRI system.

### Protocol Development

The specific contrast of the IR‐DTI sequence, which is a combination of the diffusion decay and the inversion recovery effect, was simulated using two crossing fibres, oriented along *x* and *y* axis, associated with *T*_1_s of 800 and 1000 ms, respectively. The fibres had identical diffusion properties (diffusion parallel to the fibre *D* = $1.3\times 10^{-3}{\text{mm}}^{2}/s$), but had different volume fraction (0.2 and 0.3, respectively). 100000 noisy repetitions were generated adding Rician noise at different levels, so that for each fibre a mean and standard deviation was obtained. Three protocols were tested: 1 protocol 1: a scheme suitable for the crossing fibre phantom, where the fibre orientation is known a priori (so that high angular resolution is not needed), the SNR is high ( ≈20:1) and there are no time constraints (so that multiple TI can be acquired); 2 protocol 2: a scheme with higher angular resolution, suitable for fixated brain tissue with SNR ≈ 15:1; and 3 protocol 3: a fast scheme suitable for *in vivo* acquisitions (acquisition time ≈ 2 h), where SNR ≈ 8–10:1. The results of the simulations were used to prove that the three schemes could succeed in resolving two different *T*_1_s; the protocols were then applied to acquire experimental data as described in the next session.

### Data Acquisition

A phantom consisting of freshly excised porcine optic and sciatic nerves was built placing the two nerves perpendicularly one above the other, immersed in a proton‐free fluid (Fluorinert FC‐77). The IR‐DTI sequence was used to image the fibre crossing sample with the following parameters: TI = 175, 250, 300, 350, 400, 450, 500, 585, 675, 750, 850, 1100, 1500 ms, 15 noncollinear gradient orientations plus two unweighted scans for each TI, *b* = $1000\;s/{\text{mm}}^{2},\text{TR}/\text{TE}=7500/20\text{ms}$. Matrix size and resolution were 72 × 96 × 2 and 0.27 × 0.27 × 3.00 mm, respectively. The whole acquisition lasted 9 h. A perfused rat brain, immersed in Fluorinert FC‐77, was scanned using the IR‐DTI sequence with the following parameters: TI = 200, 325, 450, 500, 650, 1000 ms, 30 gradient orientations 21, *b* = $1000\;s/{\text{mm}}^{2},\text{TR}/\text{TE}=3500/20\;\text{ms}$. A CHARMED protocol 6 was also acquired using 90 gradient orientations distributed over three shells of *b*‐values 1000, 2000, and 4000 s/mm^2^. Matrix size and resolution were 64 × 96 × 21 and 0.30 × 0.30 × 0.45 mm, respectively. The whole acquisition lasted 16 h. Three male Wistar rats underwent the IR‐DTI protocol *in vivo* according to the following parameters: TI = 200, 270, 400, 600, 1000 ms, 15 gradient orientations, *b* = 1000 s/mm^2^, TR/TE = 3750/30 ms. A CHARMED protocol was also acquired using the same parameters reported above. Matrix size and resolution were 96 × 96 × 15 and 0.27 × 0.27 × 1.00 mm, respectively. The whole acquisition lasted 2.45 h.

### Data Preprocessing

Eddy current, motion, and distortion corrections were performed adapting the UNDISTORT tool 22 to handle the combined *T*_1_‐diffusion contrast.

### Data Processing

The IR‐DTI signal was modeled as a combination of two fibre populations (A and B), each characterized by a volume fraction, *f_i_*, a specific diffusion tensor *D_i_* and a specific $T_{1}^{i}$, according to the following formula:

(1) $$S/S0=f_{A}\times (1-2\times \exp(-\text{TI}/T_{1}^{A}))\times \exp(-b\times D_{A})+$$

$$f_{B}\times (1-2\times \exp(-\text{TI}/T_{1}^{B}))\times \exp(-b\times D_{B})$$

For the fibre crossing phantom, *f_A_*, *D_A_*, *D_B_*, $T_{1}^{A}$, and $T_{1}^{B}$ were free parameters in the Levenberg–Marquardt fit, and *f_B_* = 1 − *f_A_*. For brain samples, the CHARMED model was previously used to fit *f_A_*, *D_A_*, *f_B_*, and *D_B_*, leaving only the two *T*_1_s ( $T_{1}^{A}$ and $T_{1}^{B}$) as free parameters. This allows the method to be applied to arbitrary fibre systems without the need for a high angular resolution in the IR‐DTI data that would result in excessive scanning times (as a complete diffusion data set would need to be acquired at each of the TIs).

The CHARMED analysis was done using an in‐house fitting routine written in Matlab (The Mathworks, Natick, MA). This is a modification of the original protocol proposed in 5, adapted to fit the data acquired with a high field scanner. The fitted orientations were also used to perform tractography using ExploreDTI software (Leemans, 2009). Fibre tracts were extracted in the rat brain using waypoints to virtually dissect the corpus callosum and the cingulum in each hemisphere.

Standard *T*_1_ maps were computed according to $S/S0=1-2\times \exp(-\text{TI}/T_{1}^{s})$ (where the superscript *s* stands for single) using only the nondiffusion‐weighted inversion recovery signals.

### Analysis in Standard Space

The correlation between diffusion tensor MRI, CHARMED and *T*_1_ metrics was evaluated in 35 ROIs obtained from the intersection of the FA‐derived skeleton from the tract‐based spatial statistic pipeline 23 and standardized WM labels in standard space, according to the method described in 24, adapted to handle animal data.

## RESULTS

### Simulations

**Figure 1:**
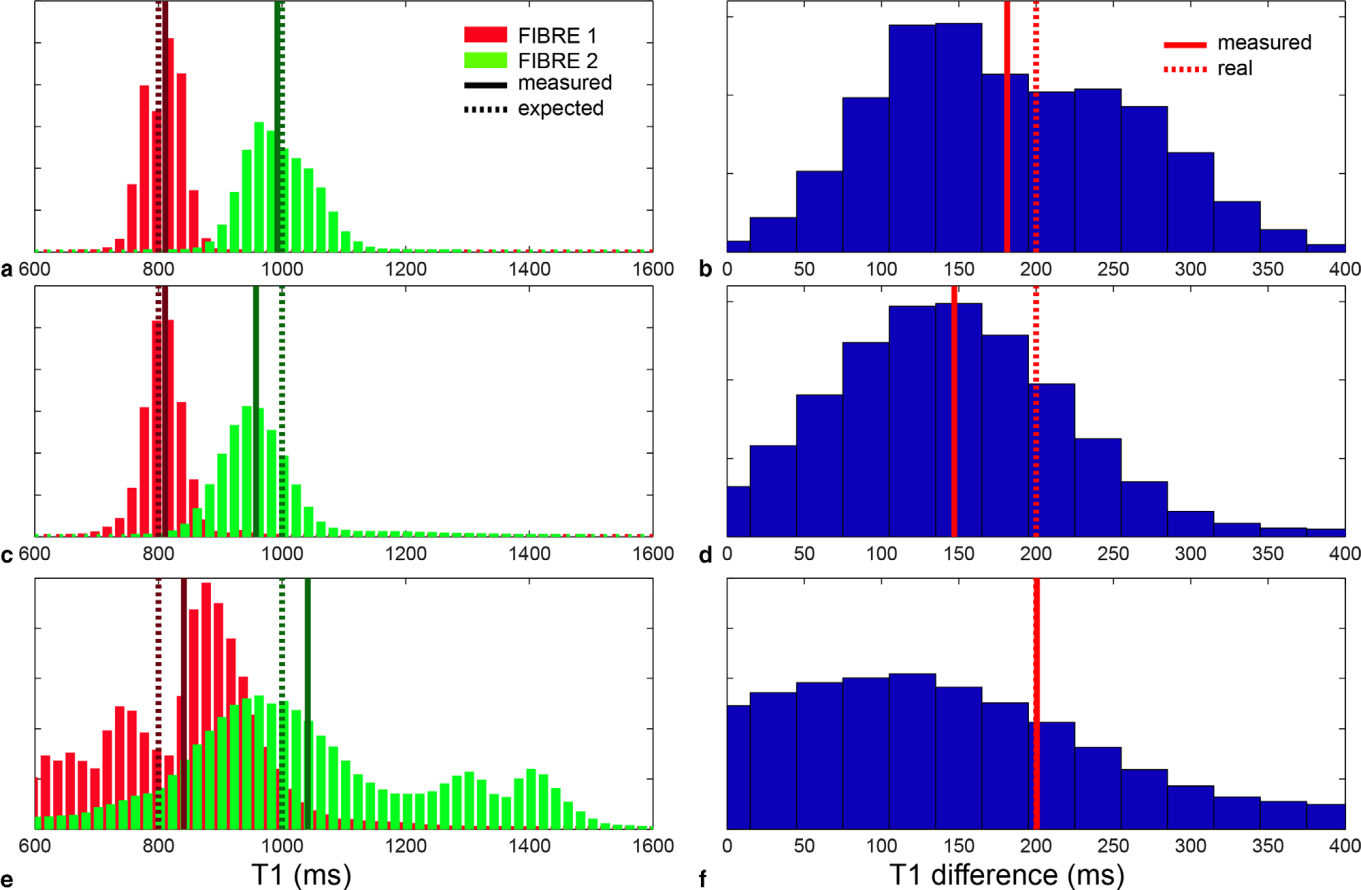
**a**: Histograms of the two *T*_1_ components for protocol 1, suitable for acquisition on phantom. **b**: Histogram of the difference between the two *T*_1_s for protocol 1. **c**: Histograms of the two *T*_1_ components for protocol 2, suitable for acquisition on fixed brain. **d**: Histogram of the difference between the two *T*_1_s for protocol 2. **e**: Histograms of the two *T*_1_ components for protocol 3, suitable for acquisition *in vivo*. **f**: Histogram of the difference between the two *T*_1_s for protocol 3. In all the histograms, dashed lines are expected values, continuous lines are measured values.

Preliminary simulations were used to test the capability of the method to disentangle the two components with different experimental conditions. Figure 1a shows the histogram of the two *T*_1_s for protocol 1, where the two peaks are clearly separated. Figure 1b shows the difference between the two components. Similarly, Figure 1c,d shows the histograms for protocol 2, and Figure 1e,f shows the histograms for protocol 3. Even with more challenging experimental setups, the analysis succeeds in disentangling the two components correctly. The standard deviations of the *T*_1_s are 5% and 10% for protocol 1, 13% and 18% for protocol 2, and 16% and 23% for protocol 3, calculated for the lower and higher *T*_1_, respectively.

### Fibre Crossing Phantom

**Figure 2:**
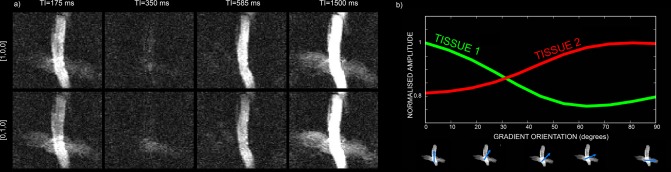
**a**: Raw data acquired on the fibre crossing phantom, consisting of freshly excised porcine optic and sciatic nerves, for two gradient orientations: [1 0 0], that is, parallel to the fibre oriented along the *x* axis, and [0 1 0], that is, parallel to the fiber oriented along the *y* axis. The data are shown for four different TIs: 175, 350, 585, and 1500 ms. **b**: Normalized weights of the two fibres including the volume fraction and the diffusion contribution, plotted for different applied gradients ranging from [1 0 0] to [0 1 0].

The fibre crossing phantom, comprising two excised porcine nerve fibers with very different myelination, was used to compare the ability of the method in areas of crossing fibres with results using standard relaxometry. Figure 2a shows the raw data acquired on the fibre crossing phantom for two gradient orientations: [1 0 0], that is, parallel to the fibre oriented along the *x* axis, and [0 1 0], that is, parallel to the fiber oriented along the *y* axis. The data are shown for four different TIs. Due to the different orientation of the fibres with respect to the applied gradient, the signal is differentially attenuated by diffusion for the same TI. This is further illustrated in Figure 2b, where the normalized weights of the two fibres are plotted for different applied gradients ranging from [1 0 0] to [0 1 0].

**Figure 3:**
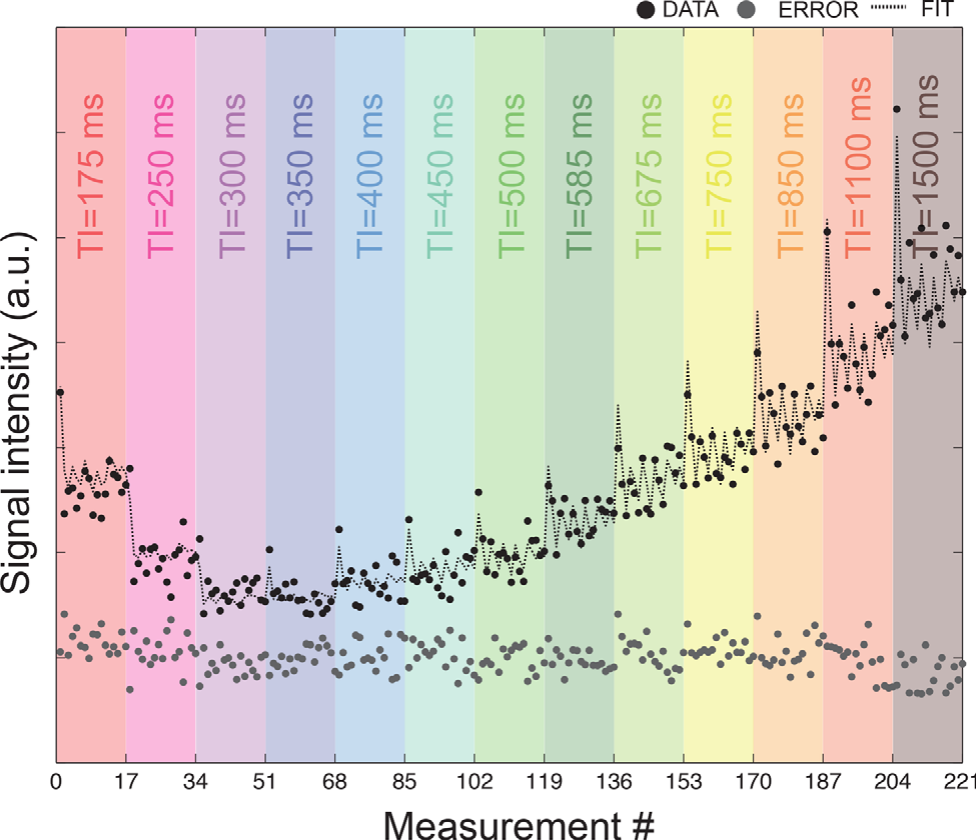
Experimental data (black circles), fit (dashed line), and error (gray circles) for one representative voxel in the crossing finer phantoms. Datapoints are reported for increasing TIs, each in a different color.

Figure 3 shows the fit to the raw data at varying diffusion orientations and TIs for one representative voxel in the crossing area. The trend is a combination of the diffusion decay with the typical inversion recovery curve. Normality test was performed voxel‐wise on the distribution of the residuals and more than 85% of the voxels passed it.

**Figure 4:**
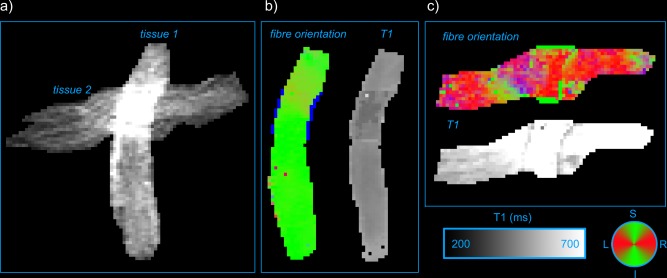
**a**: *b* = 0s/mm^2^ image of the fibre crossing phantom. **b**: Directionally encoded color map 25 and *T*_1_ map of tissue 1. **c**: Directionally encoded color map and *T*_1_ map of tissue 2.

Figure 4a shows the *b* = 0 s/mm^2^ image of the fibre crossing phantom, where the two orthogonal tissues are easily recognized. Figure 4b,c shows the results of the IR‐DTI analysis, where the two tissues show clearly different fibre orientations and different *T*_1_s.

**Figure 5:**
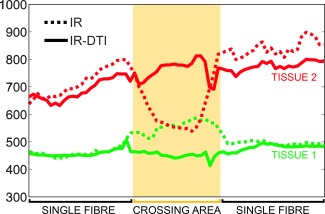
*T*_1_ profiles along the two tissues in the fibre crossing phantom, calculated using conventional inversion recovery (dotted line) and IR‐DTI (continuous line). In yellow, the area where the two tissues cross.

Figure 5 shows the *T*_1_ profiles along the two tissues, calculated using conventional inversion recovery and IR‐DTI. The profiles were calculated using the method described in 26. In the crossing area, conventional inversion recovery fails to recover two distinct *T*_1_s and returns instead an intermediate value of *T*_1_, which is the weighted contribution of the *T*_1_s of each fibre.

**Figure 6:**
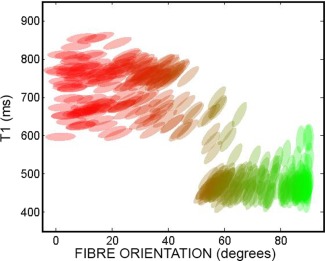
Scatterplot of *T*_1_ values versus the angle of the associated fibre with respect to the *x* axis for the fibre crossing phantom, in the area of fibre crossing. Each ellipse represents the diffusion tensor in the plane *x‐y* and is colored according to the directionally encoded color convention.

Figure 6 shows the *T*_1_ values versus the angle of the associated fibre with respect to the *x* axis. This scatterplot clearly shows that the IR‐DTI approach is able to cluster the fibres according to their *T*_1_ and diffusivity properties: fibres in the upper‐left area of the plot have higher *T*_1_ and are predominantly oriented along the *x* axis while fibres in the lower‐right area of the plot shows lower *T*_1_ and are predominantly oriented along the *y* axis.

### Perfused Rat Brain

**Figure 7:**
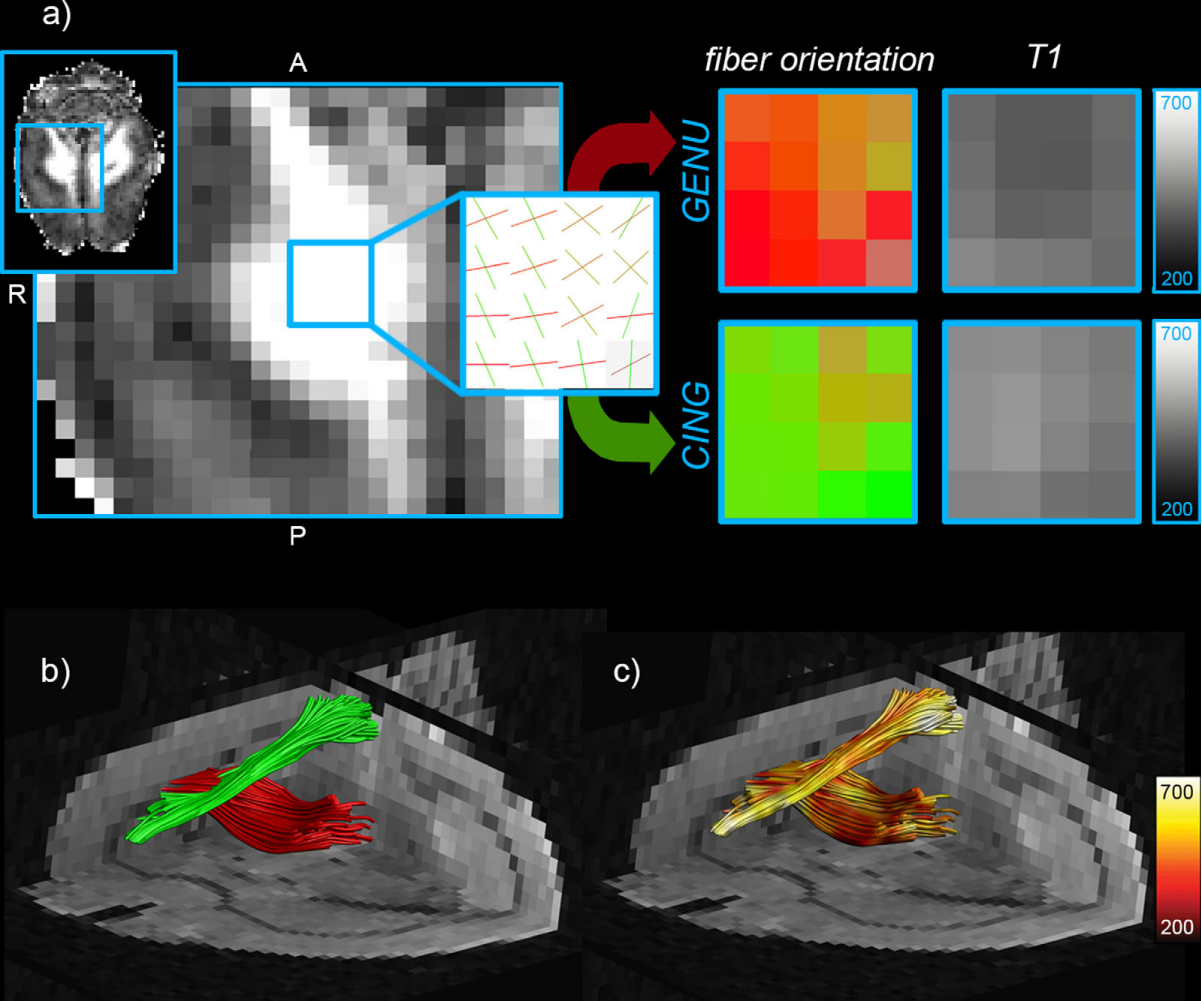
Perfused rat brain: **a**) Restricted volume fraction map and zoom on an area where the cingulum bundle and the corpus callosum cross. The CHARMED analysis extracts the two different orientations and the IR‐DTI approach assigns a different *T*_1_ for each fibre. **b**: Tractography reconstruction of the corpus callosum (red) and the cingulum (green). **c**: *T*_1_ maps projected onto the two fibre bundles. All *T*_1_ values are expressed in ms.

Due to the limited anatomical resolution of the scan, the cingulum bundle and the genu of the corpus callosum effectively cross within a voxel, providing a good test‐bed for IR‐DTI in the brain. CHARMED analysis successfully recovered two distinct orientations in the crossing area (red and green) and the associated *T*_1_ maps show different *T*_1_ values for each fibre population, as shown in Figure 7. The tractometry analysis 27 on the two bundles returned *T*_1_ = 456 ms for the genu and *T*_1_ = 494 ms for the cingulum.

### *In Vivo* Rat Brains

This cohort of live rats was used to replicate the results obtained in the perfused brain using a protocol feasible *in vivo* and to investigate correlation between the fitted parameters.

**Figure 8:**
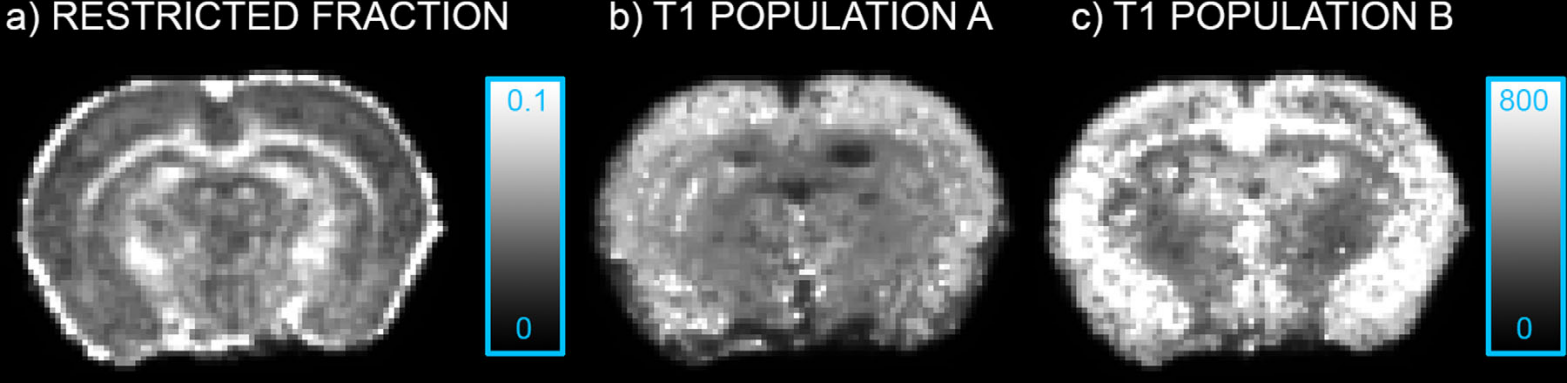
**a**: Total restricted volume fraction for one of the *in vivo* rat brain acquired; **b**: map of the *T*_1_ population associated with the larger restricted fraction; and **c**: map of the *T*_1_ population associated with the smaller restricted fraction. All *T*_1_ values are expressed in ms.

Figure 8 shows the maps of the total restricted fraction (a) and *T*_1_ maps associated to the two populations (b and c, where population A is the one associated with the higher restricted fraction) for one of the three *in vivo* rat brains. Similar patterns were seen for the other two animals. The population associated with the higher restricted fraction has higher *T*_1_ in the cortex and the gray matter while it shows smaller *T*_1_ in the WM.

**Figure 9:**
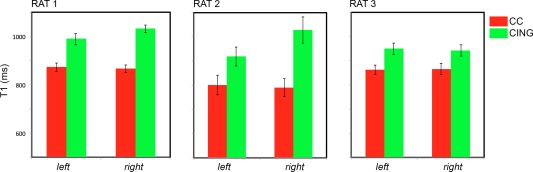
*T*_1_ values and associated standard errors calculated in an area of crossing between the cingulum and the corpus callosum for the three rats *in vivo*.

The tractometry analysis on the two bundles, cingulum and corpus callosum, was repeated *in vivo*, and the difference between the *T*_1_ values in the crossing regions are reported in Figure 9. The cingulum consistently shows higher *T*_1_ than the corpus callosum on all scanned brains and in both hemispheres.

### Correlation Between Restricted Fraction and T_1_

**Figure 10:**
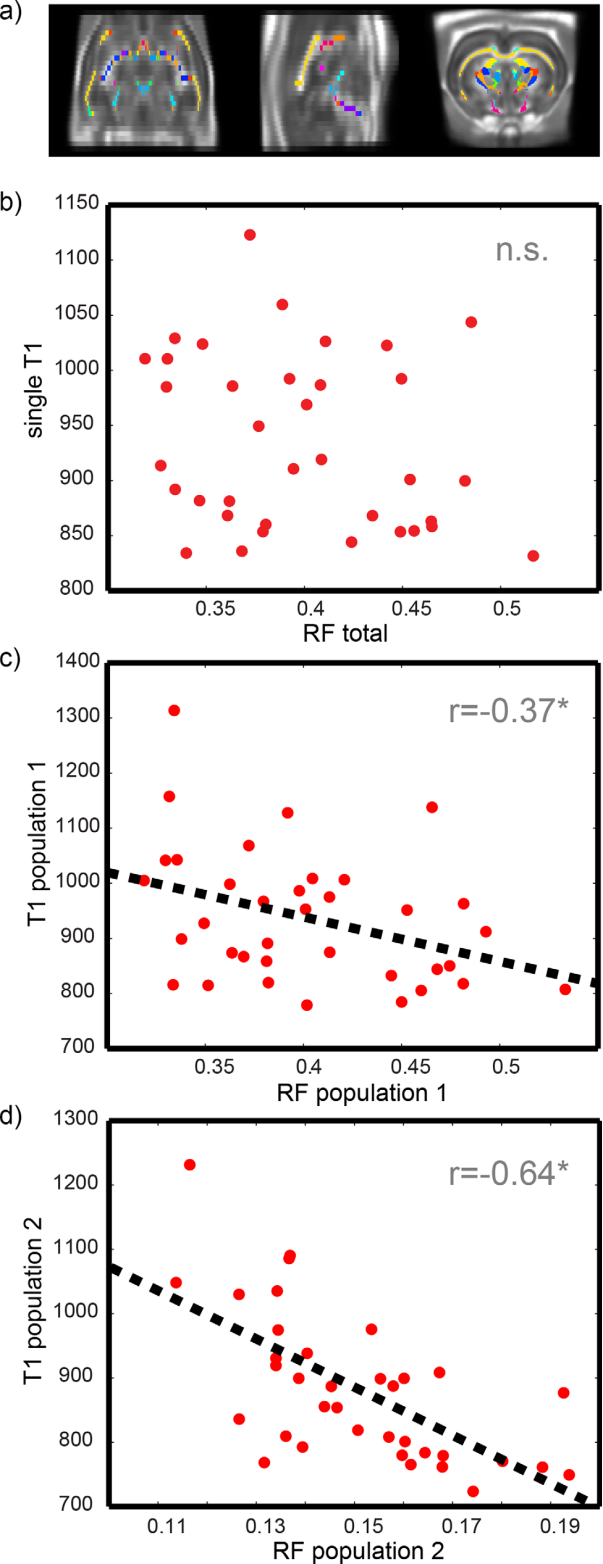
*In vivo* rat cohort: **a**) WM mask combined with WM parcellation, superimposed on the FA template. **b**: Correlation plot between single *T*_1_ and the total restricted volume fraction. **c**: Correlation plot between *T*_1_ and restricted fraction of population 1. **d**: Correlation plot between *T*_1_ and restricted fraction of population 2

Figure 10 shows the normalized rat brain template and the correlation between *T*_1_ and the restricted fraction from CHARMED. The correlation between a single *T*_1_, obtained using conventional inversion recovery, and the total restricted fraction from CHARMED is not significant at *P* = 0.001 (Fig. 10b), while if two different *T*_1_s are fitted in each voxel, and each one is correlated with its own restricted fraction, then a significant correlation is found as shown in Figure 10c,d.

## DISCUSSION

To tackle the lack of specificity of conventional MRI methods to the different fibre populations present within a voxel, we propose to combine inversion recovery and diffusion acquisitions to exploit the orientational dependence of the diffusion decay and gain orientation‐specific information on *T*_1_. This method effectively provides, in each voxel, fibre‐specific values of *T*_1_ and restricted fraction, allowing to tease out the contribution of the fibre architecture when assessing multivariate WM properties.

The method is tested using simulations and then applied to different systems. The results on the fibre crossing phantom show that the method succeeds to recover the *T*_1_ of each fibre while conventional inversion recovery only provides an average *T*_1_ value in the crossing region. Note that the results on the fibre crossing phantom can be considered a validation of the method, as the myelination of each fibre was known a priori. We did not attempt to use conventional validation (i.e., histology) to validate the measurements since methods to assess myelin are, to the best of our knowledge, unable to obtain a fibre‐specific measurement when fibres cross with each other. The only way of validating our technique was then to artificially superimpose in the same voxel two objects with different myelination patterns and try to resolve them.

The results on the rat brain provide a convincing evidence that differences in *T*_1_ between two crossing bundles can be consistently recovered, even without prior knowledge of the local fibre architecture. In the crossing areas between the corpus callosum and the cingulum, a consistent difference between the *T*_1_ associated with the two bundles is measured. This result could be explained by the fact that the corpus callosum is more myelinated than the cingulum [see 28 on developing humans and 29 in the rhesus monkey] and myelin content is inversely correlated with *T*_1_ 30. In addition, the results show that when two components are recovered in each voxel, then there is a significant correlation between axonal and relaxometry features (Fig. 10c,d). The correlation is in agreement with the results reported by 26, where significant correlations between *T*_1_ and axonal features are found only in areas of single fibre populations. Our IR‐DTI method now allows recovery of this correlation also in areas of fibre crossing.

The potential applications of this technique are numerous and far‐ranging. For example, different authors have reported unusual increase of fractional anisotropy in the presence of disease selectively affecting one fibre bundle in crossing fibre areas. Reference 31 reported almost no change in diffusion anisotropy in WM pathways undergoing Wallerian degeneration, only in regions where the degenerated pathway crosses other tracts, such as in the rostral pons. Reference 32 reported a preferential loss of connections along specific radiating directions in Huntington's disease. The same group 33 explained the increase of fractional anisotropy in Alzheimer's disease by a relative preservation of motor‐related projection fibres at the early stage of the disease. We speculate that in such cases, the technology proposed here would provide further insight into the disease mechanisms by being able to characterize relaxometry and axonal properties along each of the constituent fibre bundles within the voxel. In addition, due to the new ability to make measurements that are specific to single fibre populations (yielding increased sensitivity to changes), we believe this method can detect very early alterations of the WM in crossing fibre areas that might not be detected using other MRI‐based approaches to quantifiying *T*_1_.

Potential applications are not limited to diseases but include the study of WM normal development, aging, and plasticity. For example, approaches based on IR‐DTI may well lead to the ability to make estimates of the *g*‐ratio (i.e., the ratio of the inner to outer diameter of myelinated axons) across the entirety of the WM. The *g*‐ratio is believed to be a promising biomarker for WM characterization and has been also involved in explaining sex differences in microstructure 34. Methods to estimate the *g*‐ratio using MRI so far rely on the assumption of a single fibre population 35 and can thus be applied only in areas of coherent fibre orientation like the corpus callosum.

The IR‐DTI framework proposed here is a first comprehensive proof of concept of the method. The translation to human is the subject of on‐going work. The sequence can be implemented without the need for sequence programming as most MRI vendors provide the ability to add an inversion pulse to the start of a sequence. Possible limitations for human *in vivo* application are the long acquisition times that would result due to elevated SAR. This can be fixed, for example, by implementing state of the art SAR‐efficient inversion pulses 36.

We further note that our current implementation of IR‐DTI always fits two fibre populations. This might lead to suboptimal precision in areas characterized by a single dominant fibre population, and incorrect results where more fibre orientations are needed, or in gray matter, where capturing the underlying architecture can be challenging due to lower anisotropy. This could explain the hyperintensity in the T1 maps of population B of Figure 8. A refinement would be to introduce a model selection step 37 to decide voxel‐wise which is the most appropriate number of fibers to take into account. In addition, the framework could be trivially extended to more comprehensive relaxometry models 11. These issues will be addressed in a future work.

Although for the crossing fibre phantom it is safe to assume that the differential *T*_1_ contrast is due to differences in myelination, for more complex systems like the brain WM more caution is needed. A correlation between *T*_1_ and myelin content has been extensively reported in literature 29, 38, 39, 40, 41, 42, and demyelinating pathologies are known to produce changes in *T*_1_ 43, 44, 45. However, changes in total *T*_1_ relaxation properties of WM arise from multiple water pools, not just myelin, confounding accurate assessment of myelin by relaxometry 46. Specifically, *T*_1_ contrast is also sensitive to factors like edema, gliosis, and axon density. This is a possible confound that should be taken into account and will be also addressed in future work.

To conclude, our method resolves both diffusion and relaxometry properties in the presence of crossing fibres, obtaining tract‐specific values of the restricted fraction and *T*_1_.
